# A sequencing-based method for quantifying gene-deletion mutants of bacteria in the intracellular environment

**DOI:** 10.3389/fmicb.2024.1487724

**Published:** 2025-01-28

**Authors:** Xiao Fei, Zengzhi Yuan, Sandra Marina Wellner, Yibing Ma, John Elmerdahl Olsen

**Affiliations:** ^1^Key Laboratory of Animal Pathogen Infection and Immunology of Fujian Province, College of Animal Sciences, Fujian Agriculture and Forestry University, Fuzhou, China; ^2^Department of Veterinary and Animal Sciences, Faculty of Health and Medical Sciences, University of Copenhagen, Copenhagen, Denmark; ^3^Tianjin Key Laboratory of Animal and Plant Resistance, Tianjin, China; ^4^College of Life Sciences, Tianjin Normal University, Tianjin, China

**Keywords:** next-generation sequencing, intracellular survival, gene-deletion mutants, *Salmonella*, bacteria

## Abstract

Advancements in next-generation sequencing (NGS) have significantly accelerated the development of innovative methodologies in microbiological research. In this study, we present a novel method to quantify the net survival of gene-deletion mutants within the intracellular environment. Based on standardized Illumina short-read sequencing of genomic DNA, the method eliminates the need for specific selective markers on each deletion mutant. For validation, the method was shown to accurately quantify mutants in spiked pools of mixed mutants, showing no statistically significant differences compared to the expected values based on CFU determination (*p* > 0.05). Further, the method was used to quantify mutants of *S*. Gallinarum in macrophages. Six mutants and one control strain were mixed in a pool and allowed to infect HD11 cells for 2 h. The results align with prior research results, providing evidence of the feasibility of mixed mutant infections in functional gene identification. Notably, the simplicity and standardization of the method, rooted in standard whole-genome sequencing protocols, make it easily implementable across various laboratories.

## 1 Introduction

Genomic editing methods are fundamental tools for scientists to investigate and understand the biological functions of genomic elements in bacteria (Nakashima and Miyazaki, 2014; Arroyo-Olarte et al., 2021). Through site-specific deletions, researchers can investigate the roles of specific genes in processes such as multiplication and metabolism and assess their contributions to pathogenicity using *in vitro* and *in vivo* virulence assays. Advancements in genomic editing techniques continue to expand our understanding of bacterial physiology, pathogenicity, genetics, and their implications for disease and treatment strategies (Cain et al., 2020; Holden and Hensel, 1998; Langridge et al., 2009; García et al., 2022).

In the case of gene-deletion mutants, the use of pools of mutants offers several advantages in studies of pathogenicity. It parallelizes the assessment of fitness effects caused by gene deletions and significantly reduces the number of experimental animals needed in *in vivo* experiments, aligning with the 3R framework, which advocates for reduced use of experimental animals (Törnqvist et al., 2014; Tannenbaum and Bennett, 2015). Using a mixed-mutant infection strategy thus contributes to more ethical research practices, provided this can be done with robust assessment of the fitness of the individual mutant. In screening investigations, this can conveniently be done using libraries of random mutants, such as in the Transposon Directed Insertion Sequencing (TRADIS) technique (Langridge et al., 2009; García et al., 2022). However, in the case of work with defined deletion mutants, no similar technique is available.

To distinguish among different defined, gene-deletion mutants, researchers genetically incorporate specific markers. A common strategy is to introduce specific antibiotic resistance genes into different mutants and subsequently quantify them by culture methods based on their antibiotic resistance. With advancements in DNA sequencing technology, marker DNA sequences can also serve as barcodes for quantitative real-time PCR (qPCR) analysis, offering an alternative way to quantify different mutant strains within a pool of mixed mutants (Coward et al., 2008; Winderl et al., 2008; Yoon et al., 2011; Grant et al., 2008). However, adding genomic markers and establishing distinct qPCR reactions for each mutant can be labor-intensive, qPCR methods are challenging to standardize (Taylor et al., 2019), and there is a limit to the number of suitable marker genes. Thus, there is a need for a simple, standardized method for quantification of defined deletion mutants if these are to be quantified in infection studies with mixed pools of mutants.

*Salmonella enterica* serovar Gallinarum only infects avian species, causing fowl typhoid (*S*. Gallinarum biovar Gallinarum) or pullorum disease (*S*. Gallinarum biovar pullorum), both of which mostly manifest as systemic infections (Barrow and Neto, 2011). The exact mechanism behind their host specificity and high ability to cause systemic infection is unknown. Genome comparisons to the evolutionarily close, broad-host-range serovar., *S*. Enteritidis, have revealed high frequency of pseudogene formation and prophage acquisition in *S*. Gallinarum, and a high number of conserved SNPs in coding and regulatory elements of shared genes, suggesting that these traits may contribute to the differences in host range and infection outcomes (Barrow and Neto, 2011; Langridge et al., 2015; Thomson et al., 2008; Fei et al., 2020). Moreover, comparative transcriptome analysis between *S*. Gallinarum and *S*. Enteritidis has shown specific differences in gene expression patterns between the two serovars in the avian hosts (Fei et al., 2023).

The aim of the current study was to develop a sequencing-based method to quantify marker-free, defined deletion mutants in the intracellular environment of cells. For validation of this methods, we selected genes of *S*. Gallinarum for mutation and studied their importance for intracellular survival in macrophages. The quantification method is generally applicable to the quantification of deletion mutants of bacteria in growth assays in culture medium, cultured cells, and potentially *in vivo* animal models.

## 2 Materials and methods

### 2.1 Bacterial strain and culture conditions

*Salmonella gallinarum* biovar Gallinarum (denoted *S*. Gallinarum throughout the manuscript) strain G9 (Barrow et al., 1994) was used as the wild-type strain. Strains were routinely cultured in Luria-Bertani (LB) medium or LB agar plates (Oxoid, Denmark) at 37°C, except for the strains containing the temperature-sensitive plasmid pKD46, which were grown at 30°C. When necessary, the medium was supplemented with gentamicin (GEN; 20 μg/mL) or chloramphenicol (CHL; 25 μg/mL). Antibiotics were obtained from Sigma (Sigma, Denmark).

### 2.2 Construction of gene-deletion mutants

The reference genome of *S*. Gallinarum G9 (GenBank accession no. CM001153) was used as the template for designing primers. Site-specific gene deletions were carried out with the *λ* Red mutagenesis protocol with minor modifications (Datsenko and Wanner, 2000). In brief, the primers with gene specific overhangs (sequences provided in Supplementary Table S1) were used to amplify the CHL resistance cassette from the DNA template of plasmid pKD3. The competent *S*. Gallinarum G9 strain, which contains the plasmid pKD46, was induced with arabinose during preparation. The purified PCR product was then transformed into the competent cells via electroporation. Subsequently, the electroporated bacteria were allowed to recover in S.O.C. Medium (Thermo Scientific, USA) at 30°C for 2 h statically. Cultures were plated on LB plates with CHL and incubated overnight at 42°C. Finally, CHL resistant colonies were PCR verified for gene deletions as previously described (Datsenko and Wanner, 2000).

### 2.3 Preparation of pools of deletion mutants

*S*. Gallinarum G9 gene-deletion mutants were cultured individually in LB broth overnight. Cultures were adjusted to an optical density at 600 nm (OD_600_) to yield 1 × 10^8^ CFU/mL, which was confirmed by plating of 10-fold serial dilutions on LB agar plates and counting CFU after overnight incubation at 37°C. For initial testing of the ability of the method to quantify mutants, three pools of four mutants with a designed composition of 1:1:1:3 were prepared. The approximate CFU concentration was confirmed by plating on LB agar plates, as described above. Three hundred μL of each pool was spread onto LB agar plates containing 25 μg/mL CHL and incubated at 37°C for 16 h. The resulting bacterial colonies were collected by washing with PBS, centrifuged at 12000 rpm for 5 min, and pellets were stored at −20°C for later use.

### 2.4 Macrophage infection assay

Cell infection experiments were conducted as previously described, with minor modifications (Huang et al., 2019; Fei et al., 2023). Briefly, HD11 chicken derived macrophages were cultured in RPMI 1640 medium (Gibco, Denmark) with 10% fetal bovine serum (FBS; Biowest, Denmark) at 37°C in 5% CO_2_. Before infection, cells were seeded into 24-well plates at a density of 2 × 10^5^ per well, and further culturing for 16 h to reach approximately 80% confluence. Individual *S*. Gallinarum deletion mutants were cultured overnight and adjusted to an approximately equal density at 600 nm (OD_600_). Equal volumes of deletion mutants were combined, and the resulting mixture was then adjusted to an appropriate concentration of 5 × 10^6^ CFU/mL and added to cultured macrophages at a multiplicity of infection (MOI) of 10:1. Plates were centrifuged at 1000 rpm for 10 min to promote interactions between bacteria and cells (time point, 0 h). After 1 h of incubation, the cells were washed twice with Dulbecco’s Phosphate Buffered Saline (DPBS) and then incubated in RPMI 1640 medium containing 100 μg/mL GEN and 10% FBS for an additional 1 h to kill the extracellular bacteria (time point 2 h). The wells were then washed twice with DPBS and lysed with 0.1% Triton X-100. The lysed mixtures in each well were adjusted to a suitable dilution and spread onto LB agar plates containing 25 μg/mL CHL and incubated at 37°C for 16 h. The bacterial colonies from pools of mutants were washed with PBS and centrifuged at 12000 rpm for 5 min. The pellets were stored at −20°C for later use.

### 2.5 DNA extraction and sequencing

Genomic DNA was extracted from the bacterial pellets using the GenElute™ Bacterial Genomic DNA Kit (Sigma-Aldrich, USA). The quality and quantity of the extracted DNA were assessed using the NanoDrop™ One C (Thermo Scientific, USA). Double-stranded high molecular weight DNA with an OD 260/280 ≥ 1.8–2.0 and containing >200 ng/μL was considered suitable for library preparation. A standard genomic Illumina 150 bp paired-end library was generated from the isolated genomic DNA according to the instructions from the supplier. The sequencing process was carried out using Illumina NovaSeq 6,000 sequencing technology at Eurofins Genomics (Constance, Germany). The raw reads and data generated for this study are available in the NCBI Sequence Read Archive under BioProject PRJNA1112735.

### 2.6 *In silico* analysis

#### 2.6.1 Mapping of reads

Reads were mapped using a custom made Snakemake (Mölder et al., 2021) pipeline, available at https://github.com/china-fix/rq-count_v1. Initially, raw reads in fastq format were preprocessed by fastp version 0.12.4 (Chen et al., 2018) to obtain clean reads. Subsequently, clean reads were aligned to the reference genome (in this study the reference genome was *S*. Gallinarum G9, accession no. CM001153) using Burrows-Wheeler Aligner (BWA) version 0.7.17 (Li and Durbin, 2009). Duplicate reads were removed using Picard Toolkit version 2.18.7 (Broad Institute). Finally, samtools version 1.17 (Li et al., 2009) was used to count and output the depth of reads at each position of the reference genome.

#### 2.6.2 Calculation of quantity of individual deletion mutants

**Figure 1:**
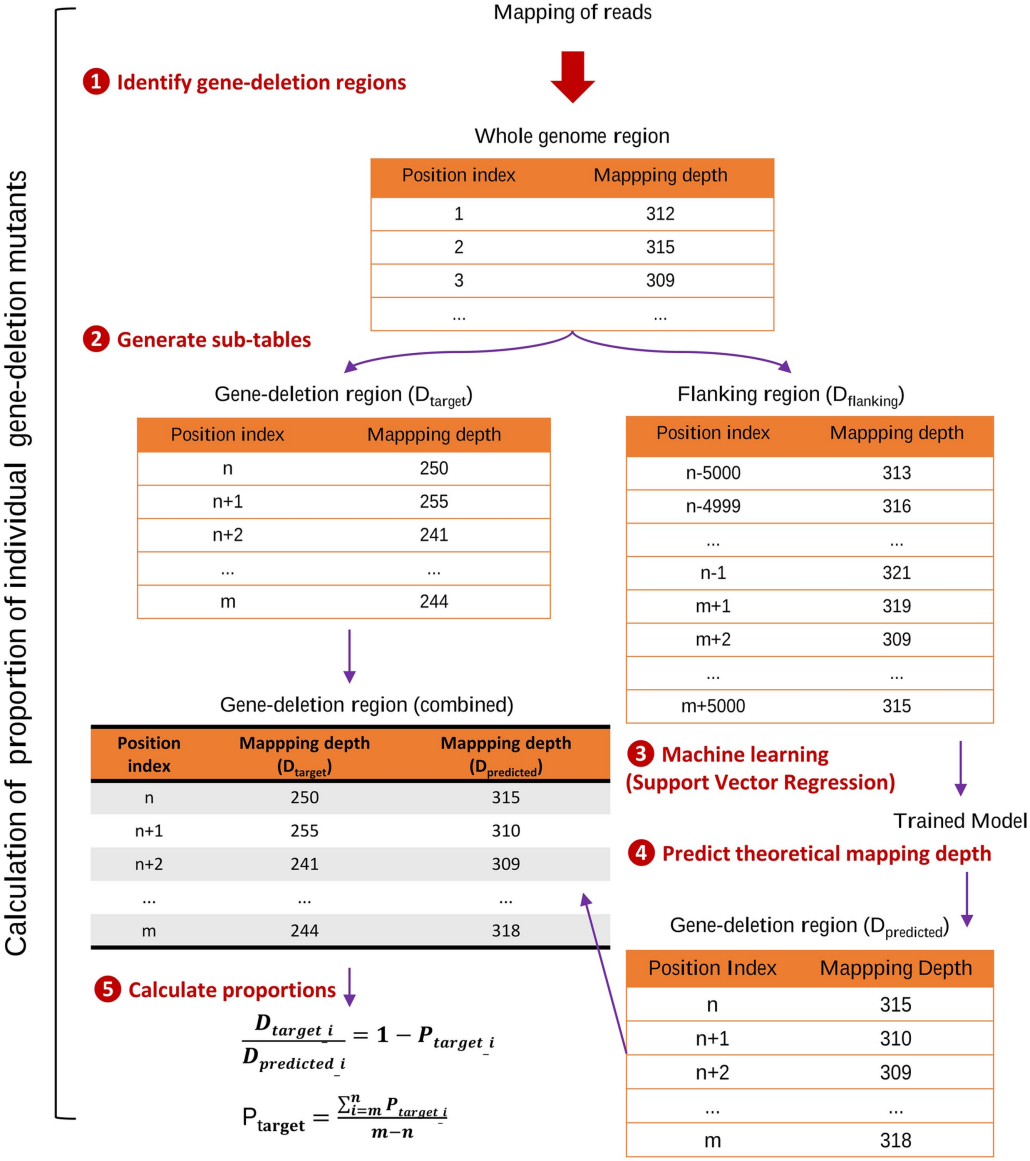
Calculation of quantities of marker-free gene-deletion mutants in infection experiments using pools of deletion mutants. The figure illustrates the key steps involved in calculating the quantity of gene-deletion mutants using a Python script. (1) Identification of gene-deletion regions within the reference genome, forming the basis for subsequent analysis. (2) Based on sequence data, two sub-tables were extracted: D_target_, recording the mapping depth of each gene-deletion region; and D_flanking_, capturing the mapping depth for the flanking regions of each deletion. (3) Machine learning: the mapping depth data from the D_flanking_ table was utilized to train a Support Vector Regression model. (4) Predict theoretical mapping depths: the trained model predicted mapping depths (D_predicted_) for each gene-deletion region, simulating scenarios where the deletion mutant still retains the deletion region in the mixed sample, where all other mutants still carried the specific region. (5) Calculate quantifies of mutants: the program then calculated the difference between the actual mapping depths (D_target_) and the predicted depths of the gene-deletion region (D_predicted_). This difference served as a measure of the proportion of each mutant strain within the mixed sample (P_target_).

The quantity of mutant strains was calculated based on the output file with data on the depth of sequencing from above, using a custom made program run in Python 3.11 environment,^1^ available at https://github.com/china-fix/rq-count_v1. The steps involved in the calculation process are summarized in Figure 1. Briefly, the program first read the mapping depth for the entire reference genome. It then identified the positions of the different gene-deletion regions and extracted two sub-tables: one recording the mapping depth of each gene-deletion region (D_target_) and the other the mapping depth for the flanking region (D_flanking_). By default, the flanking region was set to cover 5,000 bps upstream and downstream of the deleted region. Data from the flanking region table was fed into a Support Vector Regression (SVR) model, whose output was used to predict the theoretical mapping depths at each gene-deletion region (D_predicted_), had the gene-deletion not been introduced in the specific site (i.e., similar to all other mutants in the pool that were not mutated at this site). The program then calculated the difference between the actual mapping depths (D_target_), and the predicted depths of the gene-deletion region (D_predicted_). This difference served as a measure of the relative quantity of each mutant strain within the mixed sample (P_target_).

#### 2.6.3 Calculation of proportion of individual deletion mutants

The method developed offers two ways of calculation of the proportion/quantity of individual deletion mutants. The first of these compares the outcomes of various experimental sets or biological repetitions to each other using an internal control strain. The strain used carries a deletion in the *malXY* gene, which is a pseudogene in *Salmonella,* and the deletion of which does not affect survival within the intracellular environment (Grant et al., 2008). The normalization process involved dividing the P_target_ value of each individual deletion mutant by that of a *malXY* gene deletion. This approach enabled us to effectively calibrate results across multiple experimental runs, and results are expressed as a virulence index.

For cases where such an internal control strain may not be available, we developed a control free scaling method to normalize proportion of mutants across different sets. This involved identifying the median proportion value in each experimental set, and calculating a scaling factor by taking the reciprocal of the median value. Subsequently, this scaling factor can be applied to adjust all proportion values (each value is multiplied by the scaling factor) within the set. This approach ensures that the median value becomes 1 across all different sets, while other values are appropriately scaled. The approach and its pitfalls are described in details in https://github.com/china-fix/rq-count_v1. The results of this analyses are indexes of fitness.

### 2.7 Statistical analysis

To compare the relative proportions of mutants based on culture and sequencing methods in the initial proof of concept, the proportions, as determined by viable counts, were transformed into expected mapping depths for each gene-deletion region. This was achieved using the actual sequencing depth for its compared observed group in the corresponding sequencing run (Supplementary Table S2). Mapping depths for each gene-deletion region were calculated as described above using the script available at https://github.com/china-fix/rq-count_v1. Expected and observed mapping depths were rounded to the nearest integer and compared using the chi-square (χ^2^) test. Statistical analyses were performed using GraphPad Prism version 8.3.0 (GraphPad Software). *p-*values ≤0.05 were considered statistically significant.

## 3 Results

### 3.1 Overview of the quantification analysis workflow

**Figure 2:**
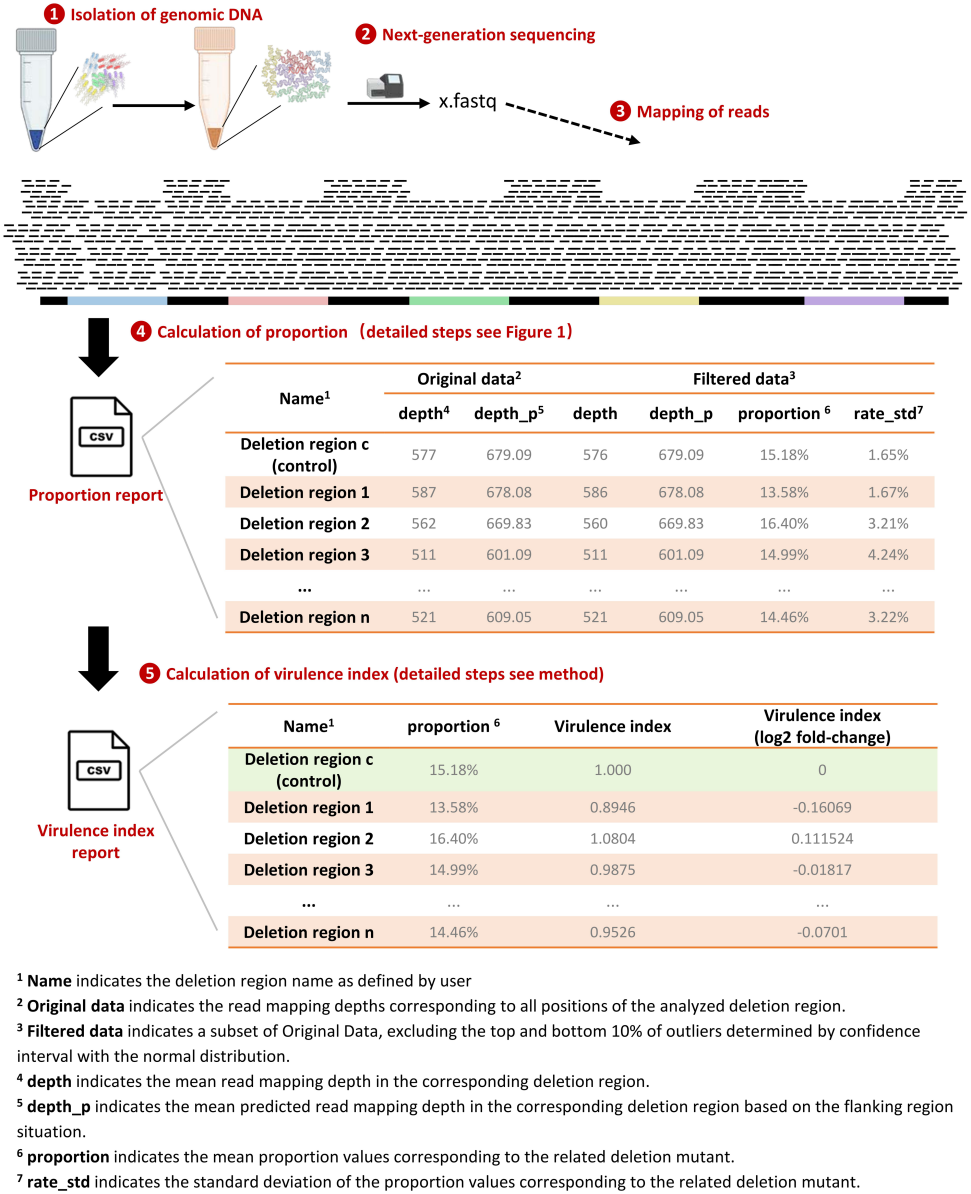
Workflow for quantification of bacterial gene-deletion mutants. The workflow includes five steps: (1) Isolation of genomic DNA: Genomic DNA was extracted from a mixture of different gene-deletion mutants, including a fully virulent strain with a pseudogene deletion; (2) Next-generation sequencing; (3) Mapping of reads: Raw reads were mapped to the reference genome of the wild-type strain using a custom pipeline; (4) Calculation of the proportion of mutants: The proportion of bacteria lacking the deleted genomic regions was quantified based on reduced mapping depth in these regions; (5) Calculation of the virulence index: Normalization by dividing the proportion of each mutant with the number of the fully virulent mutant to obtain a growth or virulence index.

As shown in Figure 2, the quantification workflow consists of five main steps, with steps 1–4 compiled into automated scripts that summarize the results into a CSV file. The optional step 5 involves scaling the proportion of individual deletion mutants based on a control strain.

1. Genomic DNA was extracted from broth or macrophages containing a mixture of gene-deletion mutants derived from the same wild-type strain.
2. The isolated genomic DNA was subjected to next-generation sequencing.
3. The raw sequencing reads were mapped to the reference genome of the wild-type strain using a custom-made script in the Snakemake pipeline. Detailed steps are described in material and methods section.
4. The proportion of each deletion mutant in the pool was determined using a custom-made program in Python. This utilizes the fact that deletion mutants lacked a region in the sequence, and therefore, the mixed samples show reduced sequence depth in this region. Detailed steps are described in material and methods section.
5. Scaling the proportion of individual deletion mutants based on a control strain (virulence index). Detailed steps are described in the material and methods section.

The proportion report is a CSV file recording the mapping depths and calculated proportion for each deletion targets. As shown in the example in Figure 2, the CSV file includes ‘Filtered Data,’ a subset of the ‘Original Data,’ excluding the top and bottom 10% of outliers determined by a confidence interval with a normal distribution. Comparing the differences in depth values between these two datasets reflects the influence of outliers. An ideal mapping depth distribution in the related deletion region would be uniform and conform to a normal distribution; therefore, outlier filtering will have minimal impact on the median mapping depth, as evidenced by the example of results in Figure 2. The summary report also includes the standard deviation of the proportion values for each deletion mutant, providing insights into the variability of these proportions in a single sequencing run.

### 3.2 Influence of deletion target length and the usage of its flanking region length on the accuracy of *in silico* proportion calculations

**Figure 3:**
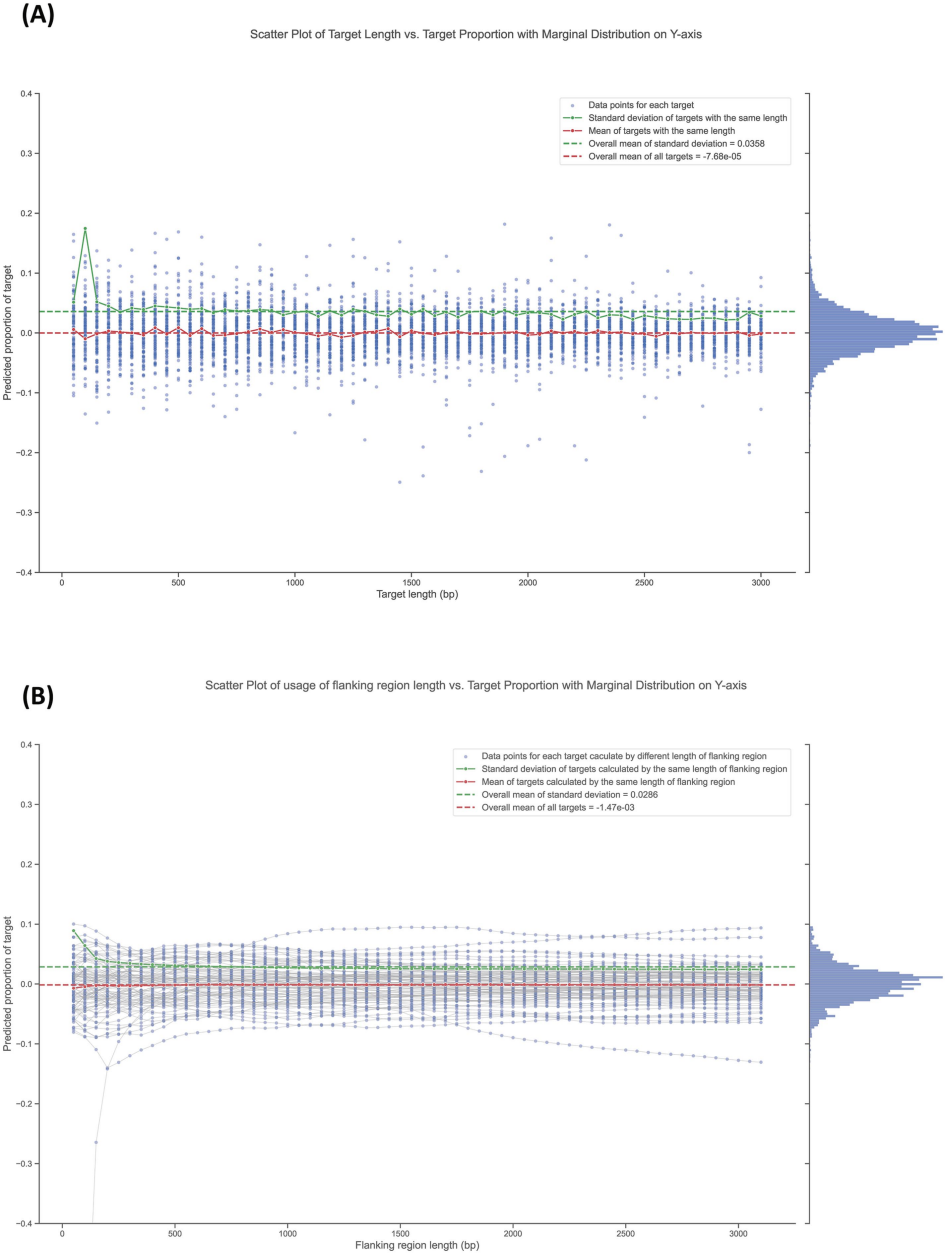
Influence of deletion target length and the usage of its flanking region length on proportion calculations. **(A)** Scatter plot of target length vs. target proportion with marginal distribution on the Y-axis. Data points represent individual target lengths, ranging from 50 bp to 3,000 bp, tested at 50 bp intervals. Each target length was evaluated 100 times by randomly selecting different targets of the corresponding length. **(B)** Scatter plot of flanking region length vs. target proportion with marginal distribution on the Y-axis. Data points (blue points) represent the predicted proportion of targets (3,000 bp in length) calculated using flanking region lengths from 50 bp to 3,000 bp. The grey lines connect data points calculated using different flanking region lengths for the same target.

Before validating the sequencing-based quantification method in practical applications, we first simulated multiple deletion targets *in silico* to evaluate the appropriate gene-deletion lengths and the usage of flanking region lengths in the workflow. Whole genome sequencing data from pure wild-type bacterial culture was used as the testing data. Because there are no deletion mutants in pure wild-type bacterial culture, the predicted proportion of each target under every length of flanking region usage should theoretically be 0. As shown in Figure 3A, we tested target lengths from 50 bp to 3,000 bp at 50 bp intervals, repeating the proportion prediction for each target length 100 times by randomly selecting different targets of the corresponding length. The overall mean of all targets was −7.68 × 10^−5^, which is very close to the expected value of 0, while the overall mean standard deviation was 0.0358. Negative mean values lack biological meaning, but may arise in calculations, when the measured target proportions are very low (near 0), such as when a deletion mutant is effectively excluded during competition. In these cases, random noise might cause the predicted sequencing depth to slightly exceed the measured depth in the target sequence region.

Generally, standard deviation values for the same length are higher than average at short target lengths less than around 400 bp. Above this length, the values stabilize and remain at a lower level. Figure 3B shown the prediction of target proportions (target with length of 3,000 bp) calculated using flanking region lengths from 50 bp to 3,000 bp. Data points calculated using different flanking region lengths (blue points) for the same target were linked using a grey line. The overall mean of all data points was −1.47 × 10^−3^, which is very close to the expected value of 0. The overall mean standard deviation was 0.0286. Generally, standard deviation values of data points calculated using the same flanking region length are higher than average at short lengths less than around 800 bp. After that, the values stabilize and remain at a lower level. Moreover, we found that some targets consistently received predicted proportion values far from the theoretical value of 0. This *in silico* test was repeated with three different sequencing datasets from pure wild-type bacterial culture, each yielding similar results (see Supplementary Figure S1).

### 3.3 Construction of *Salmonella* Gallinarum mutants for method validation and case study application

To validate the sequencing-based quantification method in practical applications, we chose the avian-specific *Salmonella* strain *S*. Gallinarum G9 as the wild-type backbone strain. We constructed 14 gene-deletion mutants for this study. These mutants were used to assess the quantitative reliability of our new method and to evaluate the intracellular survival of mutants after challenging macrophages with pools of mixed bacterial mutants.

As depicted in Table 1, the 14 mutants contained deletions in conserved genomic differences between the host-specific serovar *S*. Gallinarum and its evolutionarily close, broad host-range serovar., *S*. Enteritidis. The ∆*prgH* and ∆*ssaT* mutants represent disruptions in Type III secretion system 1 (T3SS-1) and Type III secretion system 2 (T3SS-2), respectively. To include a control strain for comparison in the mixed sample, we constructed a pseudogene deletion mutant with deletion of the genes *malXY* (∆*malXY*). The deletion region used for this mutant was previously employed for wild-type isogenic tagging in *Salmonella* and not shown to influence virulence (Grant et al., 2008).

**Table 1:** *Salmonella* Gallinarum mutants constructed and used for validation of quantification method in this study.

Strain name	Short name	Location of mutation^1^	Target gene and size^2^
∆*malXY*::CHL^R^	∆*malXY*	1,730,094…1,730,648	Pseudogenes *malX* and *malY*, 555 bp
∆*pgtE*::CHL^R^	∆*pgtE*	2,496,007…2,497,045	Outer membrane protease E, 1039 bp
∆*sbmC*::CHL^R^	∆*sbmC*	2,129,882…2,130,449	DNA gyrase inhibitor, 568 bp
∆*stfA*::CHL^R^	∆*stfA*	232,474…233,134	Fimbrial major subunit, 661 bp
∆*steB*::CHL^R^	∆*steB*	1,559,869…1,560,370	Type III secretion system effector SteB, 502 bp
∆*sscB*::CHL^R^	∆*sscB*	1,781,100…1,781,585	SycD/LcrH family type III secretion system chaperone, 486 bp
∆*leuO*::CHL^R^	∆*leuO*	134,306…135,350	Transcriptional regulator, 1,045 bp
∆PART1::CHL^R^	∆PART1	1,284,969…1,297,434	Encodes components of prophage gifsy-2, about 12.4 kb
∆PART2::CHL^R^	∆PART2	1,268,761…1,275,220	Encodes prophage like components, about 6.4 kb
∆PART4::CHL^R^	∆PART4	327,820…334,737	Encodes 7 CDSs, about 6.9 kb
∆PART5::CHL^R^	∆PART5	306,318…318,540	Encodes 21 CDSs, about 12.2 kb
∆SPI-19::CHL^R^	∆SPI-19	1,118,782…1,147,177	Part of SPI-19, about 28.3 kb,
∆*prgH*::CHL^R^	∆*prgH*	2,891,806…2,892,984	Cell invasion protein, component of T3SS-1 needle-like complex, 1,179 bp
∆*ssaT*::CHL^R^	∆*ssaT*	1,768,625…1,759,404	T3SS-2 apparatus protein SsaT, 780 bp

^1^ Location of mutation indicates the start and end position of the deleted region indexed by the reference sequence (GenBank accession no. NC_011274). ^2^ Target gene and size give a brief explanation of the annotation of deleted region and the size of the deleted region.

### 3.4 The workflow can accurately quantify the relative proportions of mutants in mixed pools

**Figure 4:**
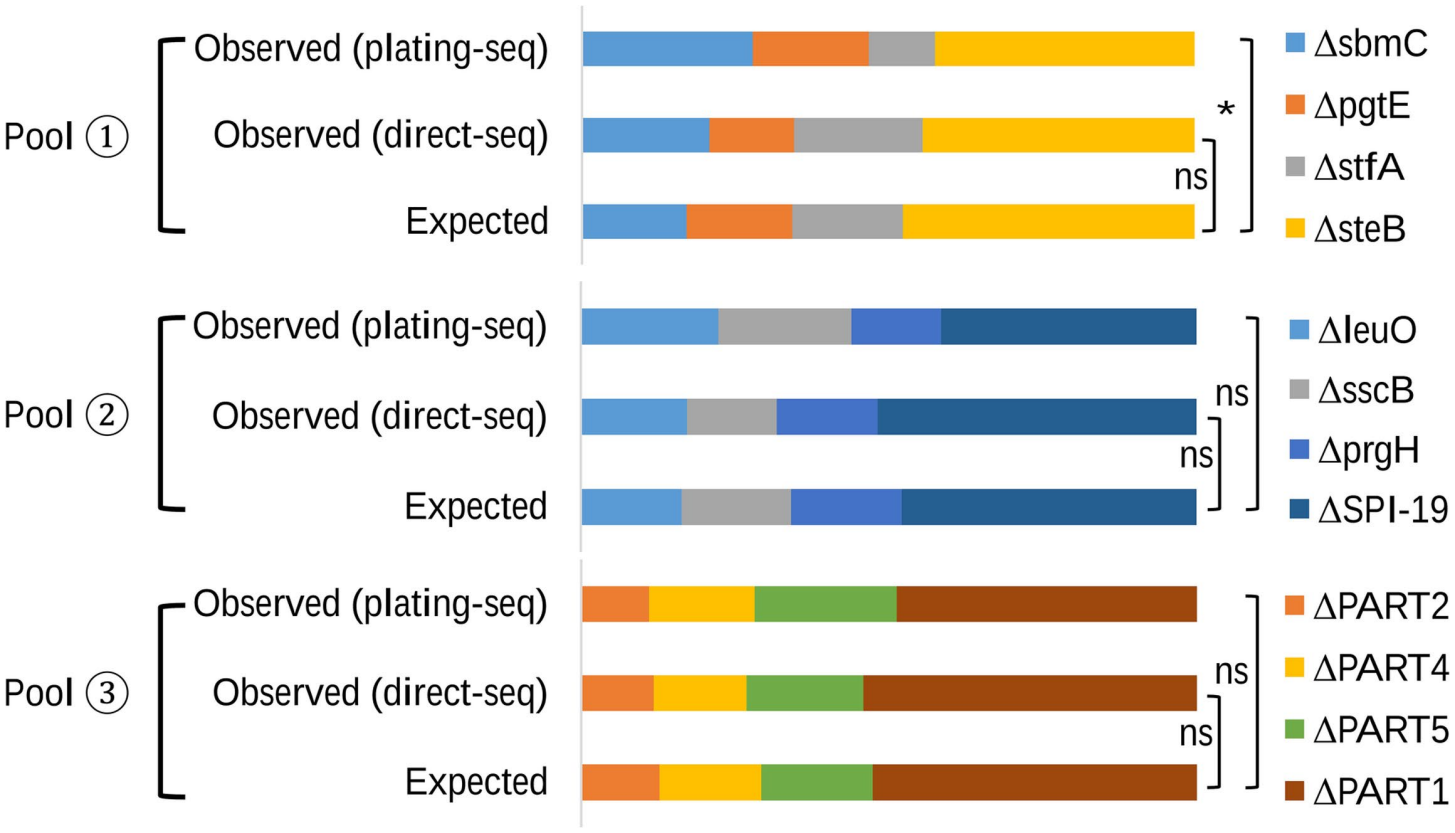
The relative proportion of mutants in each mixed pool. The Expected group represents the pre-designed proportions of mutants, validated by plating and CFU counts. The Observed (direct-seq) group represents proportions obtained through the novel sequencing-based quantification method, with DNA was isolated directly from the mixed sample. The Observed (plating-seq) group quantifies mutant proportions using genomic DNA extracted from the mixed mutant pools after plating on LB agar to control for growth-induced biases.

To assess the ability of the sequencing-based quantification method, we first created three mutant pools with known compositions. We then determined the relative proportions of each mutant using the sequencing-based method and compared these to the expected values based on CFU determination (Figure 4 and Supplementary Table S2). The Expected values corresponding to the pre-designed relative proportions of each mutant, and this was confirmed by retrospective plating and viable counts, which did not differ significantly from the Observed (direct-seq) values from the sequencing-based quantification analysis for any of the three pools analyzed (pool 1, *p* = 0.6155; pool 2, *p* = 0.7626; pool 3, *p* = 0.7890). Furthermore, to assess whether enrichment of pools on LB agar prior to sequencing introduced any growth rate related bias, an Observed (plating-seq) group was included. This group quantified the relative proportions of mutants in mixed pools after plating on LB agar.

In samples from pool 2 and pool 3, the comparison between the Expected proportion and the proportion determined by Observed (plating-seq) showed no statistically significant differences (pool 2, *p* = 0.53; pool 3, *p* = 0.81). However, the *p* value of the comparison from pool 1 was 0.04, suggesting that differences in growth rate of mutants prior to sequencing introduced bias in the analysis in this comparison.

### 3.5 Quantification of selected *Salmonella* Gallinarum deletion mutants within the intracellular environment of macrophages

**Figure 5:**
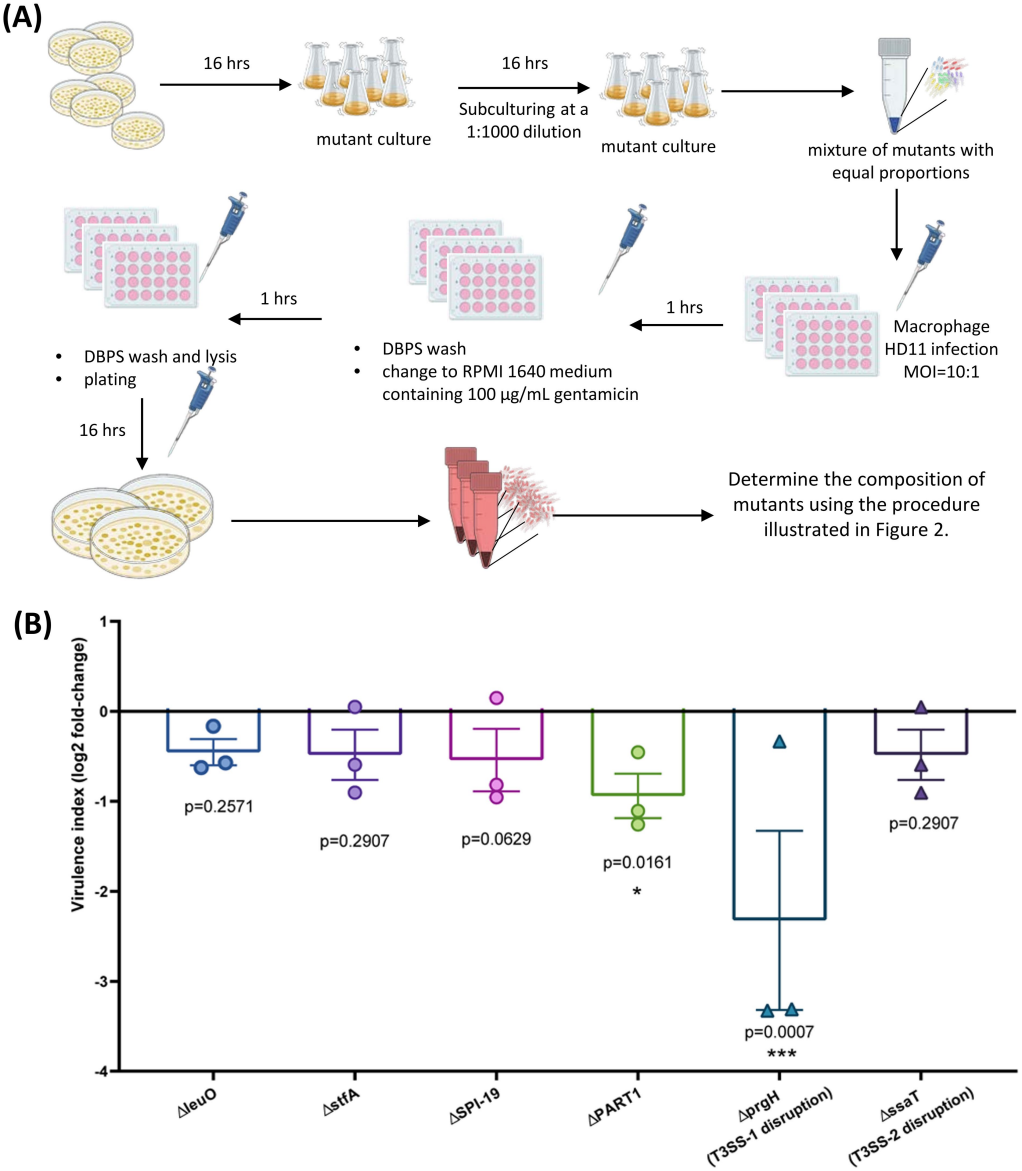
Sequencing-based method for quantifying gene-deletion mutants of bacteria in the intracellular environment. **(A)** Experimental design for macrophage infection: Pools of *S.* Gallinarum mutants were mixed in equal proportions and used to challenge macrophages at a multiplicity of infection (MOI) of 10:1. Two hours post-infection, cells were lysed, and intracellular bacteria were recovered on LB agar. Bacterial colonies obtained from the plates were scraped, and the composition of mutants was determined using the sequencing-based method. **(B)** The relative fold-change of the *S.* Gallinarum mutants 2 h post-infection compared to the control strain. Bar graphs represent the means of three separate experiments, with error bars indicating standard deviations. Statistical significance was determined by one-way analysis of variance (ANOVA) with Dunnett’s multiple comparisons test. *, *p* < 0.05; ***, *p* < 0.001.

To demonstrate the potential applications of our novel sequencing-based quantification method in research, we conducted a cell infection experiment. As illustrated in Figure 5A, the method was used to assess the uptake and survival of six *S*. Gallinarum mutants in chicken-derived HD11 macrophages over a 2-h period. A bacterial mixture with equal proportions of mutants was prepared and adjusted to challenge macrophages at a multiplicity of infection (MOI) of 10:1. Two hours post-infection, the cells were lysed, and the intracellular bacteria were recovered on LB agar. Bacterial colonies were scraped from the plates, and the composition of mutants was determined using the sequencing-based method.

Figure 5B summarized the relative fold-change of the *S*. Gallinarum mutants 2 h post-infection compared to the control strain with gene knockout in the pseudogenes *malXY*, providing a virulence index relative to wild type virulence. The ∆*prgH* mutant, lacking the gene encoding component of T3SS-1 needle-like complex, and the mutant with deletion of the unique genomic region present in *S*. Pullorum but not *S*. Enteritidis, PART1 (Fei et al., 2020), displayed a significantly down-regulate virulence index, with log2 fold-change values of −2.32 (*p* = 0.007) and − 0.94 (*p* = 0.0161), respectively. The other test mutant also showed a down-regulated index, but this was not statistically significant.

## 4 Discussion

In this study, we developed a novel *in silico* workflow to quantify the relative abundance of pools of bacterial gene-deletion mutants within the intracellular environment of cells. To demonstrate its applicability, we used this workflow in a practical case study to evaluate the survival of *S*. Gallinarum mutants within macrophages. This approach provides a simple and efficient method for studying bacterial interactions with host cells and assessing gene function in pathogenicity and survival.

Before validating the sequencing-based quantification method in practical applications, we simulated multiple deletion targets *in silico* to evaluate appropriate gene-deletion lengths and the usage of flanking region lengths in the workflow. As shown in Figure 3, longer prediction targets demonstrated greater accuracy in multiple tests, indicated by a lower standard deviation than the average. According to our tests, deletion targets longer than around 400 bp are best suitable for detection with the method. Similarly, we evaluated the influence of the usage of flanking region lengths on prediction accuracy. Our tests indicate that usage of flanking regions longer than 800 bp are recommended. This finding also indicates that genomic regions spaced less than 800 bp apart present challenges for our method. Thus, the method may not be suitable for quantification of mutants less than this size, as well as mutant with overlapping sequences. Overall, these results indicate that our workflow has broad applicability.

Subsequent practical application within different mixed mutant pools with known compositions proved that our workflow accurately quantified the relative proportions of mutants in mixed samples (Figure 4). Another possible weakness of the method was revealed during this experiment, where we harvested mutants after plating and growth on agar platers. This may introduce a growth bias to the quantification (e.g., Figure 4, Pool 1). To address this limitation, it is recommended to obtain bacterial DNA directly after growth within the model system used (macrophages or live animals, for example); however, this may be challenging from a technical point of view.

Our method allows for the determination of the relative proportions of bacterial deletion mutants in mixed infections without the need for specific selective markers. It is compatible with mixture of gene-deletion mutants, where the same antibiotic resistance gene is used as marker, and it is compatible with mutants, where the antibiotic resistance gene or other sequences used for marker has been flipped out. This feature simplifies the construction of multiple gene-deletion mutants as well as downstream analysis, and it eliminates potential growth biases introduced due to the presence of different antimicrobial resistance genes in the mutants. Additionally, the number of different antibiotic resistance genes that are available for mutant construction is limited. Compared to quantitative real-time PCR (qPCR), which can also be used to quantify the relative abundance of individual strains (Coward et al., 2008; Winderl et al., 2008; Yoon et al., 2011), it does not require case-specific design and validation of different PCR reactions for each mutant, which can be labor-intensive and time-consuming.

The quantification method is based on standard Illumina paired-end library preparation and sequencing. The use of a well-established and highly standardized platform, commercially available at a relatively low cost (Goodwin et al., 2016; Mardis, 2017), simplifies implementation of the method in microbiological analysis. Other well-designed methods specifically for quantifying the relative abundance of bacterial mixtures are available (Santiviago et al., 2009; Ahn et al., 2006), however, they often require specialized or customized equipment or technology, which can limit their adoption in different laboratories. Transposon-insertion sequencing based methods, such as Tn-Seq (van Opijnen et al., 2014), TraDIS-Seq (Langridge et al., 2009) and RB-TnSeq (Wetmore et al., 2015), offer significant advantages for assessing the relative abundance of mutants in large, randomly generated mutant populations. However, these methods are expensive and technically demanding. Despite these challenges, they provide valuable insights into mutant fitness and gene function. In certain research contexts, the focus may be limited to the function of a specific subset of target genes, particularly their relative fitness. To address these practical needs, our method provides a more accessible and straightforward alternative, enabling broader adoption and application in studies of bacterial gene functions and interactions.

Sequencing depth plays a critical role in accurately determining the proportions of individual mutants within a pool with our method. While increasing sequencing depth enhances resolution, it also imposes technical and economic constraints, particularly with current next-generation sequencing (NGS) technologies (Sims et al., 2014). Consequently, although our method performs effectively for pools containing dozens of mutants, its scalability to very large mutant libraries is restricted by these sequencing depth requirements.

*S*. Gallinarum mutants were used as a case study to illustrate the method’s potential. This bacterium replicates in macrophages, evidenced by its presence in lymphatic tissues and organs during systemic infection (Barrow and Neto, 2011). Mutant strains of *Salmonella*, impaired in macrophage replication, exhibit avirulence in host infections (Fierer and Guiney, 2001). This underscores the significance of bacterial survival and replication within macrophages for disease outcomes. In this case study, we used our newly developed sequencing-based method to evaluate the relative invasion and survival abilities of six *S*. Gallinarum deletion mutants inside macrophages. The *malXY* pseudogenes have previously been used as the gene-editing region for *Salmonella* wild-type isogenic tagging due to their lack of phenotypical influence on the backbone wild-type strain (Grant et al., 2008). In the case study, we used the ∆*malXY* gene-deletion mutant as a control strain. Including such a “pseudo-wild type strain” is necessary to obtain virulence indexes of mutants, which can be compared across different experiments. The SPI-1 gene *prgH*, encoding a T3SS-1 component (Marlovits et al., 2004; Kimbrough and Miller, 2000), has previously been established as essential for *Salmonella* cell invasion (Wang et al., 2017). A significantly down-regulated virulence index of ∆*prgH* compared to the control strain was observed in the macrophage infection experiment. This confirms the functional role of *prgH* in *S*. Gallinarum, similar to other *Salmonella* serovars during cell invasion, and supports the robustness of our mixed infection model. PART1 is a homologous sequence in *S*. Gallinarum corresponding to a conserved genomic region consistently present in *S*. Pullorum but absent in strains of *S*. Enteritidis previously reported (Fei et al., 2020). It spans approximately 12.4 kb and encodes components of prophage Gifsy-2, comprising 12 putative CDSs according to the annotation of the PHASTER web server^2^ (Zhou et al., 2011; Arndt et al., 2016). This region is likely derived from horizontal gene transfer (HGT), which has been suggested to mediate the spread of bacterial virulence genes (Penadés et al., 2015). The significantly down-regulated virulence index supports its importance in *S*. Gallinarum macrophage infection. The region named SPI-19 is part of *Salmonella* Pathogenicity Island 19, encoding the type VI secretion system (Blondel et al., 2009). We also observed a down-regulated index, albeit not significant (*p* = 0.0629), in ∆SPI-19 mutant, whose deletion might also be involved in macrophage invasion (Xian et al., 2020; Schroll et al., 2019). In summary, our case study demonstrated that the novel sequencing-based quantification method can be applied to studies of bacterial interaction with macrophages. We anticipate that this approach can also be extended to *in vivo* infection models, potentially reducing the need for experimental animals, which is a priority (Törnqvist et al., 2014; Tannenbaum and Bennett, 2015).

## Data Availability

The datasets presented in this study can be found in online repositories. The names of the repository/repositories and accession number(s) can be found below: https://www.ncbi.nlm.nih.gov/, PRJNA1112735.

## Footnotes

^1^ https://www.python.org/

^2^ http://phaster.ca/
