# Supplementary material for: A sequencing-based method for quantifying gene-deletion mutants of bacteria in the intracellular environment
Source: Front Microbiol. 2025 Jan 28;15:1487724. doi: 10.3389/fmicb.2024.1487724 (PMC11841384; doi:10.3389/fmicb.2024.1487724)
Supplement: Supplementary file 1 [file Supplementary_file_1.docx]

**Supplementary materials**

**
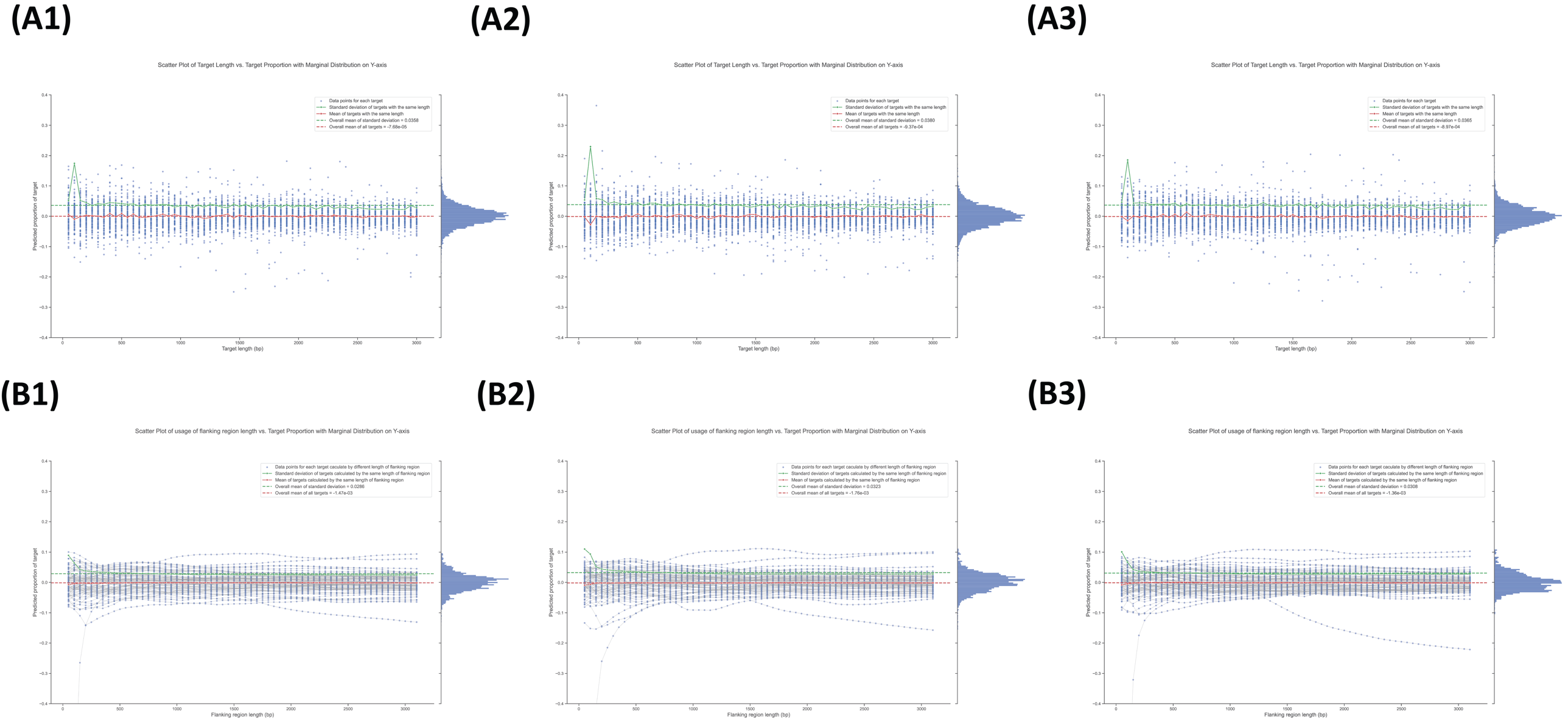
**

**Figure S1.** Influence of deletion target length and the usage of its flanking region length on proportion calculations. (A1-3) Scatter plots of target length vs. target proportion with marginal distribution on the Y-axis. Data points represent individual target lengths, ranging from 50 bp to 3000 bp, tested at 50 bp intervals. Each target length was evaluated 100 times by randomly selecting different targets of the corresponding length. (B1-3) Scatter plots of flanking region length vs. target proportion with marginal distribution on the Y-axis. Data points (blue points) represent the predicted proportion of targets (3000 bp in length) calculated using flanking region lengths from 50 bp to 3000 bp. The grey lines connect data points calculated using different flanking region lengths for the same target.

**Table S1.** Plasmids, strains and primers used for mutagenesis used in this study.

| **Plasmids, strains or primers** | **Description^*^ or sequence (5’→3’)** | **Source or comments** |
| --- | --- | --- |
| **Plasmids** |  |  |
| pKD46 | rep_pSC101_^ts^ Gen^R^ P_araBADγβ_ exo | (1) |
| pKD4 | rep_R6K_ γAmp^R^ FRT Cm^R^ FRT | (2) |
| **Strains** |  |  |
| *S*. Gallinarum G9 | wild type *S*. Gallinarum strain | (3) |
| **Primers** |  |  |
| DEL_pgtE_F | agcaaaaagtcgtcatcggccggttatgaccgatgacatcctgatgtggtgtgtaggctggagctgcttc | used for ∆pgtE mutant construction |
| DEL_pgtE_R | cctgagcatcaaattctatttcttaacttcaatataaggtaaaaatgcgtcatatgaatatcctccttag |  |
| CE_pgtE_F | caatctcccaatacttata | use as forward primer for ∆pgtE mutant RCR verification |
|  |  |  |
| DEL_sbmC_F | cggacggaacacattatccgtccgctcttttcagccaggaaaccagtcgtgtgtaggctggagctgcttc | used for ∆sbmC mutant construction |
| DEL_sbmC_R | gccgttgtcacacaccgttcaccgtcatctgaggtgtcaaaagggcgggacatatgaatatcctccttag |  |
| CE_sbmC_F | ccagtgggcattgatttgt | use as forward primer for ∆sbmC mutant RCR verification |
|  |  |  |
| DEL_stfA_F | ttaacgtgttgtttgcttcttttgaatgttgcatcggcaatttcataactgtgtaggctggagctgcttc | used for ∆stfA mutant construction |
| DEL_stfA_R | tttactttaagcattatcctgcggcagactgccgcaggatgatatttcaccatatgaatatcctccttag |  |
| CE_stfA_F | tactttatctatttgctacg | use as forward primer for ∆stfA mutant RCR verification |
|  |  |  |
| DEL_steB_F | tatctgtaggatgtggaatagcaatgccgggaaggacatggcatgacactgtgtaggctggagctgcttc | used for ∆steB mutant construction |
| DEL_steB_R | gccgacgggtagcgaaaatatcttaaccctgtgtctttccaggcttagtccatatgaatatcctccttag |  |
| CE_steB_F | gtgacattttgatttctat | use as forward primer for ∆steB mutant RCR verification |
|  |  |  |
| DEL_sscB_F | atattacttgccgctgacggaatatgaattttcatatttcgttctgttatgtgtaggctggagctgcttc | used for ∆sscB mutant construction |
| DEL_sscB_R | ataactggacagttttatccgccgagcatcttatccagcgacgtttttaacatatgaatatcctccttag |  |
| CE_sscB_F | ggatgtggcaatgagggac | use as forward primer for ∆sscB mutant RCR verification |
|  |  |  |
| DEL_leuO_F | ctctgtcagcgtctttatgttttccgaattttaacgctttccctttttctgtgtaggctggagctgcttc | used for ∆leuO mutant construction |
| DEL_leuO_R | ctaaaaagcagaataaaccagaatttgtttctgatttattctgcccggttcatatgaatatcctccttag |  |
| CE_leuO_F | ttccatgacatcaacacccat | use as forward primer for ∆leuO mutant RCR verification |
|  |  |  |
| DEL_PART1_F | ggccagcatgacataccaggcctgcgcggatgctataaacggagatgatggtgtaggctggagctgcttc | used for ∆PART1 mutant construction |
| DEL_PART1_R | agcaggttagaaagttcaatcatatttatcgatcgtttcgatcaaaaatacatatgaatatcctccttag |  |
| CE_PART1_F | cccgcagtgccgacaggtaa | use as forward primer for ∆PART1 mutant RCR verification |
|  |  |  |
| DEL_PART2_F | gtcgctcacctcagcatccccgggatgtaaagccggggaagcgcctgcatgtgtaggctggagctgcttc | used for ∆PART2 mutant construction |
| DEL_PART2_R | tctgcgcactacactggatagtaattattcattatatgaggcggttaaggcatatgaatatcctccttag |  |
| CE_PART2_F | cttcaggcgttcagcccattc | use as forward primer for ∆PART2 mutant RCR verification |
|  |  |  |
| DEL_PART4_F | ctgtggaaaattgcgaccacagccagagacatccaacaggagaatcgaaagtgtaggctggagctgcttc | used for ∆PART4 mutant construction |
| DEL_PART4_R | gggagatctttcgggttttaatcagctatttgatactatatcaaaaatatcatatgaatatcctccttag |  |
| CE_PART4_F | aagactgactctgacgctgtt | use as forward primer for ∆PART4 mutant RCR verification |
|  |  |  |
| DEL_PART5_F | tgcatcggtgtaaatcagcctgtaccggacatcagcgtgtcgattgagccgtgtaggctggagctgcttc | used for ∆PART5 mutant construction |
| DEL_PART5_R | ggacaaagcatcagctagatgtaggccatgtggatctaccaggtccacaacatatgaatatcctccttag |  |
| CE_PART5_F | ttggcgtacacggtattgc | use as forward primer for ∆PART5 mutant RCR verification |
|  |  |  |
| DEL_SPI19-F | cagcatcacatccatattttccccgtccagcagcccgttcagcagggcgagtgtaggctggagctgcttc | used for ∆SPI-19 mutant construction |
| DEL_SPI19_R | caaagaaaatacataaaatttcattaagcagggagggcttatctttatcacatatgaatatcctccttag |  |
| CE_SPI-19_F | gtagacgggcagtttgctg | use as forward primer for ∆SPI-19 mutant RCR verification |
|  |  |  |
| DEL_prgH_F | agatagcctgaccaaggtgttgccataatgacttccttatttacgttaaagtgtaggctggagctgcttc | used for ∆prgH mutant construction |
| DEL_prgH_R | gtgcggtaatctgctgctatcgagaacgacagacatcgctaacagtatatcatatgaatatcctccttag |  |
| CE_prgH_F | gctcgcggagacgatacca | use as forward primer for ∆prgH mutant RCR verification |
|  |  |  |
| DEL_ssaT_F | cttacggccatcacgtaatttcttttctgtaggctgttctgttttctcgcgtgtaggctggagctgcttc | used for ∆ssaT mutant construction |
| DEL_ssaT_R | tgttgaattatacccggcagataatgttacgagttggagagcatggttgacatatgaatatcctccttag |  |
| CE_ssaT_F | gtgggatgatagccaagac | use as forward primer for ∆ssaT mutant RCR verification |
|  |  |  |
| DEL_malXY_F | tcgccttgccgggtgtcgcgacgctgcacccggtctgcgccgaacgacgcgtgtaggctggagctgcttc | used for ∆malXY mutant construction |
| DEL_malXY_R | taatgctctggaaattttgcaaaaacggagtcattacgttgcaacttcgccatatgaatatcctccttag |  |
| CE_ssaT_F | ccatcgccattgagggtaa | use as forward primer for ∆ssaT mutant RCR verification |
|  |  |  |
| c1 | TTATACGCAAGGCGACAAGG | (2); use as reserved primer for all the mutants RCR verification |

*Relevant antibiotic resistance are indicated by^R^: GEN, gentamicin; KAN, kanamiycin; AMP, Ampicillin; CHL, chloramphenicol

**Table S2.** Expected and observed compositions of simulated gene-deletion mutants pools as illustrated in Figure 3

|  |  | Expected | Observed (direct-seq) | | | | Observed (plating-seq) | | | |
| --- | --- | --- | --- | --- | --- | --- | --- | --- | --- | --- |
|  | Mutant name | Relative richness  % | Relative richness  % | Sequencing depth ^a^ | Sequencing depth of gene-deletion region ^b^ | Sequencing depth of deletion region (expected) ^c^ | Relative richness  % | Sequencing depth^a^ | Sequencing depth of gene-deletion region^b^ | Sequencing depth of gene-deletion region (expected)^c^ |
| Pool 1 | ΔsbmC | 17.04 ± 1.88 | 20.78 ± 1.55 | 570.96 | 431 | 474 | 27.85 ± 2.02 | 578.47 | 393 | 480 |
|  | ΔpgtE | 17.27 ± 0.82 | 13.83 ± 2.50 | 585.56 | 489 | 484 | 18.96 ± 2.67 | 539.65 | 423 | 446 |
|  | ΔstfA | 18.04 ± 3.86 | 20.99 ± 2.89 | 625.99 | 470 | 513 | 10.81 ± 3.12 | 611.68 | 535 | 501 |
|  | ΔsteB | 47.65 ± 3.86 | 44.40 ± 2.89 | 495.63 | 236 | 259 | 42.38 ± 3.12 | 509.02 | 262 | 266 |
|  |  |  |  |  |  |  |  |  |  |  |
| Pool 2 | ΔleuO | 16.35 ± 0.47 | 17.26 ± 3.42 | 644.64 | 534 | 539 | 22.32 ± 2.94 | 503.65 | 391 | 421 |
|  | ΔsscB | 17.79 ± 0.05 | 14.59 ± 4.83 | 532.81 | 455 | 438 | 21.64 ± 3.95 | 398.36 | 312 | 327 |
|  | ΔprgH | 17.98 ± 0.89 | 16.39 ± 4.11 | 603.32 | 505 | 495 | 14.58 ± 3.98 | 433.28 | 370 | 355 |
|  | ΔSPI-19 | 47.88 ± 3.84 | 51.76 ± 3.66 | 556.46 | 270 | 290 | 41.46 ± 4.60 | 419.13 | 245 | 218 |
|  |  |  |  |  |  |  |  |  |  |  |
| Pools 3 | ΔPART2 | 12.72 ± 0.16 | 11.75 ± 5.26 | 485.34 | 437 | 424 | 11.00 ± 5.94 | 599.22 | 539 | 523 |
|  | ΔPART4 | 16.49 ± 1.59 | 15.10 ± 7.43 | 576.84 | 503 | 482 | 17.18 ± 6.86 | 620.35 | 523 | 518 |
|  | ΔPART5 | 18.14 ± 0.48 | 19.00 ± 4.86 | 597.18 | 501 | 489 | 23.07 ± 4.84 | 647.39 | 511 | 530 |
|  | ΔPART1 | 52.65 ± 2.05 | 54.15 ± 3.73 | 483.34 | 261 | 229 | 48.74 ± 4.09 | 592.94 | 329 | 281 |

a The actual average sequencing depth in the corresponding sequencing run

b The actual sequencing depth of the gene-deletion region in the corresponding sequencing run

c Transformed expected sequencing depth of the gene-deletion region achieved using the actual sequencing depth in the corresponding sequencing run

**References**

1. Doublet B, Douard G, Targant H, Meunier D, Madec J-Y, Cloeckaert A. 2008. Antibiotic marker modifications of λ Red and FLP helper plasmids, pKD46 and pCP20, for inactivation of chromosomal genes using PCR products in multidrug-resistant strains. Journal of Microbiological Methods 75:359–361.

2. Datsenko KA, Wanner BL. 2000. One-step inactivation of chromosomal genes in Escherichia coli K-12 using PCR products. Proceedings of the National Academy of Sciences 97:6640–6645.

3. Barrow PA, Huggins MB, Lovell MA. 1994. Host specificity of Salmonella infection in chickens and mice is expressed in vivo primarily at the level of the reticuloendothelial system. Infection and Immunity 62:4602–4610.
